# Lead biosorption and chemical composition of extracellular polymeric substances isolated from mixotrophic microalgal cultures

**DOI:** 10.1038/s41598-025-94372-9

**Published:** 2025-03-17

**Authors:** Wioleta Ciempiel, Magdalena Czemierska, Dariusz Wiącek, Marlena Szymańska, Anna Jarosz-Wilkołazka, Izabela Krzemińska

**Affiliations:** 1 Institute of Agrophysics, Polish Academy of Sciences, Doświadczalna 4, 20-290 Lublin, Poland; 2 Department of Biochemistry and Biotechnology, Institute of Biological Sciences, Maria Curie- Skłodowska University, Akademicka 19, 20-033 Lublin, Poland

**Keywords:** Exopolysaccharides, Microalgae, Mixotrophy, FTIR, Sorption, Metal removal, Microbiology, Ecology, Environmental sciences, Biomaterials, Environmental biotechnology

## Abstract

Extracellular polymers (EPS) produced by microalgae are considered an important factor in the process of biosorption of environmental contaminants. The study investigated the impact of mixotrophic cultivation of unicellular algae *Chlorella vulgaris*, *Parachlorella kessleri*, and *Vischeria magna* on the specific productivity and yield of total and soluble EPS as well as the biochemical composition and sorption properties of extracellular polymers in order to explore their potential to be used for biosorption. The results showed that the mixotrophic conditions enhanced the productivity and contributed to changes in the biochemical and monomer composition of EPS. Higher levels of total sugars, reducing sugars, protein, and phenolic compounds and reduced content of uronic acids were observed in the EPS isolated in the mixotrophic conditions. Rhamnose, xylose, mannose, glucose, and galactose were detected in the mixotrophic EPS samples. FTIR and ICP-OES were applied to characterise the structure of EPS and their role in Pb(II) removal. The results showed that the carboxyl groups and hydroxyl groups observed in the mixotrophic EPS played an important role in the Pb(II) sorption process. The EPS from the mixotrophic *C. vulgaris* cultures showed the highest potential for the removal of Pb(II) and the highest sorption capacity.

**Supplementary Information:**

The online version contains supplementary material available at 10.1038/s41598-025-94372-9.

## Introduction

The increasing commercial use of microalgal biomass results in the production of huge quantities of post-harvest media rich in extracellular polymers (EPS). EPS synthesised by microalgae are known for their bioremediation potential, especially towards heavy metal remediation^1^. In order to increase the commercial use of microalgal EPS, it is necessary to develop growth conditions that increase the amount of extracellular polymers produced. It has been found that mixotrophic growth conditions increase EPS synthesis by some species of unicellular algae^2,3^. Among the studied sources of organic carbon, the highest EPS yields were obtained in the presence of glucose^4^. Modifications of growth conditions exert an effect on the chemical composition of EPS, which may have a direct impact on the properties of these compounds^5^.

Extracellular polymeric substances can be bound to the surface of microalgal cells or secreted by these cells into the aquatic or soil environment, thus playing a role in metal sequestration^6^. Due to the presence of functional groups, EPS can effectively remove heavy metals from aqueous solutions and also immobilise these metals in the soil environment, thus limiting their availability to plants. The reduction of the bioavailability of toxic heavy metals is especially important in agricultural areas and during the pre-treatment of wastewater containing high levels of heavy metals. The sorption properties of EPS enable unicellular algae to grow in a heavy metal-contaminated environment^7^.

Metal sorption is a chemical process depending on numerous factors, e.g. sorbent properties, pH, contact time, temperature, and metal species^8^. A previous study demonstrated high Pb(II) sorption potential of autotrophically cultured *Parachlorella kessleri* and *Chlorella vulgaris* EPS and their lower potential towards Cd(II)^1^. There are few literature data explaining the relationship between EPS compounds and their role in lead sorption. Xie et al. proved the role of proteins in Cd sorption, while Naveed et al. and Lombardi et al. reported the role of acidic groups in pyruvate and uronic acids^9–11^. *P. kessleri* and *C. vulgaris* are freshwater strains known for their ability to produce EPS. *C. vulgaris* produce EPS also in mixotrophic conditions^2,14^. The ability of *P. kessleri* to grow in mixotrophic conditions has been reported^15,16^, but their EPS production potential has not been studied to date. In turn, *Vischeria magna* (formerly: *Eustigmatos magnus*) is a soil strain whose ability to synthesise extracellular polymers has not been explored^17,18^. Mixotrophic growth conditions exert an influence on the chemical composition and availability of functional groups of microalgal EPS, which can have an impact on the sorption properties of these compounds. pH affects the degree of protonation of functional groups of EPS^19^. pH is also one of the key factors in the sorption process of heavy metals due to the competition between protons and metal cations for binding sites^8^. However, there is limited information on the analysis of the structure of EPS extracted from mixotrophic microalgal cultures affected by various pH. There are no literature data on the effect of pH variation on the sorption properties towards Pb(II) ions of microalgal EPS as a remediation material isolated from mixotrophic cultures.

This study investigates the impact of the mixotrophic cultivation of unicellular algae *C. vulgaris*,* P. kessleri*, and *V. magna* on the productivity, biochemical composition, and sorption properties of extracellular polymers. To elucidate the mechanism of heavy metal sorption by microalgal EPS produced in the mixotrophic conditions and to explore their potential to be used in biosorption applications, the interactions between Pb(II) ions and EPS were investigated using Fourier transform infrared spectroscopy and optical emission spectrometry with inductively coupled plasma. The pH effect on the structure of EPS and its relationship with EPS sorption were evaluated. The results of this study showed that the mixotrophic growth conditions enhanced the content of total sugars, reducing sugars, proteins, and phenolic compounds in the tested EPS. The analysis of the monosaccharide composition revealed that hexoses were the dominant sugar components. The kinetics of lead ion sorption showed the highest Pb(II) removal potential and sorption capacity of EPS produced by *C. vulgaris* at pH 5 and 6 in mixotrophic conditions.

## Results

### Yield and specific productivity of total and soluble EPS

In the present study, the EPS productivity of *C. vulgaris*, *P. kessleri*, and *V. magna* cultured in the autotrophic and mixotrophic conditions was studied. *C. vulgaris* showed the highest EPS yield in the autotrophic conditions (compared only to the autotrophic conditions, Tukey’s test, *p* ≤ 0.05). The results presented in Table 1 show a positive effect of the mixotrophic growth conditions on biomass productivity and EPS synthesis by all the tested microalgae. In the mixotrophic growth conditions, a statistically significant increase in the EPS yield and specific productivity of all the species was observed.

The highest yield and specific productivity of total and soluble EPS in the mixotrophic conditions were observed in the *C. vulgaris* culture. The addition of glucose to the growth medium increased the total and soluble EPS specific productivity. The specific productivity of the soluble EPS was increased 8.4, 2.9, and 2.2-fold, respectively, in the cultures of *C. vulgaris*, *P. kessleri*, and *V. magna*, respectively. The lowest amount of EPS was synthesised by *V. magna.*

**Table 1:** Biomass concentration [g L^− 1^], yield [mg L^− 1^], and specific productivity [mg g^− 1^] of total and soluble EPS (± SD).

Sample	Biomass conc. [g L^− 1^]	Total EPS		Soluble EPS	
		Yield [mg L^− 1^]	Specific productivity [mg g biomass^− 1^]	Yield [mg L^− 1^]	Specific productivity [mg g biomass^− 1^]
*Cv*A	0.5 ± 0.05^a^	43.0 ± 3.74^a^	47.4 ± 5.65^bc^	14.3 ± 0.66^a^	15.7 ± 1.88^a^
*Cv*M	2.4 ± 0.07^d^	369.8 ± 20.36^d^	183.8 ± 23.48^e^	266.2 ± 9.57^d^	132.3 ± 16.62^d^
*Pk*A	0.8 ± 0.00^a^	18.9 ± 2.70^a^	25.2 ± 0.20^ab^	17.2 ± 0.41^a^	22.9 ± 0.18^ab^
*Pk*M	2.5 ± 0.20^b^	294.8 ± 19.88^c^	118.5 ± 9.74^d^	164.6 ± 6.47^c^	66.2 ± 5.44^c^
*Vm*A	1.3 ± 0.03^c^	21.1 ± 1.74^a^	16.1 ± 0.90^a^	19.4 ± 0.52^a^	15.1 ± 0.40^a^
*Vm*M	2.8 ± 0.07^b^	156.2 ± 11.39^b^	56.8 ± 1.25^c^	92.7 ± 3.77^b^	33.7 ± 0.74^b^

The superscript letters in the table indicate statistical significance of the presented results analysed by ANOVA and Tukey’s test (*p* ≤ 0.05).

### Biochemical composition of EPS

In order to determine the influence of the mixotrophic conditions on the chemical composition of the EPS, the content of total and reducing sugars, proteins, amino acids, uronic acid, amino sugars, and phenolic substances was determined. The results presented in Table 2 show higher levels of both total and reducing sugars in the EPS isolated from the microalgae grown in the mixotrophic conditions. The amount of total sugars in *Pk*M and *Vm*M was approx. 50% higher than in *Pk*A and *Vm*A. In turn, a 25% increase was observed in the *Cv*M variant. Similarly, the content of reducing sugars in *Pk*M, *Cv*M, and *Vm*M was 62%, 49%, and 45% higher, respectively, in comparison to the control samples (*Cv*A, *Pk*A, and *Vm*A). The EPS synthesised in the mixotrophic conditions had higher protein and amino sugar content as well. The changes in the amino acid content in the EPS varied between the species; however, statistically significant differences were observed only between the *Vm*A and *Vm*M samples. In the case of *P. kessleri*, the addition of glucose to the growth medium decreased the amino acid content in the EPS, compared to the control sample. The culture in the mixotrophic conditions also increased the content of phenolic compounds in the EPS of all the tested species (by 36%, 49%, and 61% in the EPS of *P. kessleri*, *C. vulgaris*, and *V. magna*, respectively). The highest content of phenolic compounds, i.e. 5.4% (54.09 µg mg^– 1^), was observed in the EPS of *Vm*M. The addition of glucose to the culture medium resulted in a reduction in the uronic acid content of all the EPS synthesised in the mixotrophic conditions. The highest concentration of uronic acid, i.e. 15.4% (154.4 mg g^– 1^) was observed in *Vm*A.

**Table 2:** Biochemical composition of EPS (weight% EPS) obtained from autotrophic and mixotrophic cultures.

Sample	Total sugar	Proteins	Phenolic compounds	Reducing sugars*	Uronic acids*	Amino sugars*	Amino acids*
*Cv*A	71.8 ± 2.4^b^	0.4 ± 0.01^a, b^	1.6 ± 0.4^b^	18.6 ± 2.8^a^	14.6 ± 0.7^b, c^	0.3 ± 0.00^a^	3.4 ± 0.3^a^
*Cv*M	90.6 ± 1.1^c^	1.2 ± 0.05^c^	2.5 ± 0.2^a^	36.6 ± 1.4^c^	13.5 ± 0.5^a, b^	0.4 ± 0.02^b^	4.1 ± 0.2^a^
*Pk*A	41.9 ± 3.2^a^	0.5 ± 0.01^a^	2.5 ± 0.2^a^	15.9 ± 1.3^a^	13.9 ± 1.6^a, b^	0.3 ± 0.01^a^	3.8 ± 0.3^a^
*Pk*M	86.5 ± 1.4^c^	1.6 ± 0.14^d^	4.9 ± 0.4^c^	41.5 ± 1.1^d^	13.4 ± 0.5^a^	0.4 ± 0.02^b^	2.8 ± 0.3^a^
*Vm*A	36.9 ± 1.2^a^	0.3 ± 0.03^b^	2.1 ± 0.1^a, b^	17.0 ± 0.7^a^	15.4 ± 0.1^c^	0.2 ± 0.01^c^	2.4 ± 0.1^a^
*Vm*M	72.4 ± 4.9^b^	0.5 ± 0.04^a^	5.4 ± 0.3^c^	30.7 ± 1.5^b^	13.4 ± 0.5^a^	0.9 ± 0.02^d^	8.9 ± 0.3^b^

The superscript letters in the table indicate statistical significance of the results analysed by ANOVA and Tukey’s test (*p* ≤ 0.05) (EPS = 1 mg mL^− 1^; *n* = 3; ± SD).

*After hydrolysis with 4 M TFA.

The analysis of monosaccharide composition of the EPS synthesised in both the autotrophic and mixotrophic conditions showed the presence of rhamnose, xylose, mannose, and galactose in all the tested EPS samples. Fructose was not detected only in the EPS obtained from *V. magna* cultured in the autotrophic conditions. Additionally, glucose was present in all the mixotrophic samples (Figure S1).

### Identification and analysis of functional groups in EPS

The EPS samples obtained from the autotrophic and mixotrophic cultures were investigated to identify functional groups. The FTIR spectrum of the EPS synthesised in the autotrophic and mixotrophic conditions shows the presence of bands at ca. 1030 cm^− 1^, 1142 cm^− 1^, 1250 cm^− 1^, 1410 cm^− 1^, 1603 cm^− 1^, 2925 cm^− 1^, and 3290 cm^− 1^. In addition, a band at 1373 cm^− 1^ and 1724 cm^− 1^ were present in the EPS synthesised by all the microalgae cultured in the mixotrophic conditions. In the mixotrophic growth conditions, a decrease in the intensity of the bands at 1650 –1200 cm^− 1^ in *Cv*M and *Pk*M (in *Vm*M, a slight decrease was observed only in the ca. 1250 cm^− 1^ and ca. 1600 cm^− 1^ bands) and an increase in the intensity of the 1142 cm^− 1^ and 1724 cm^− 1^ bands were observed in all the samples (Figure S2).

**Fig. 1:**
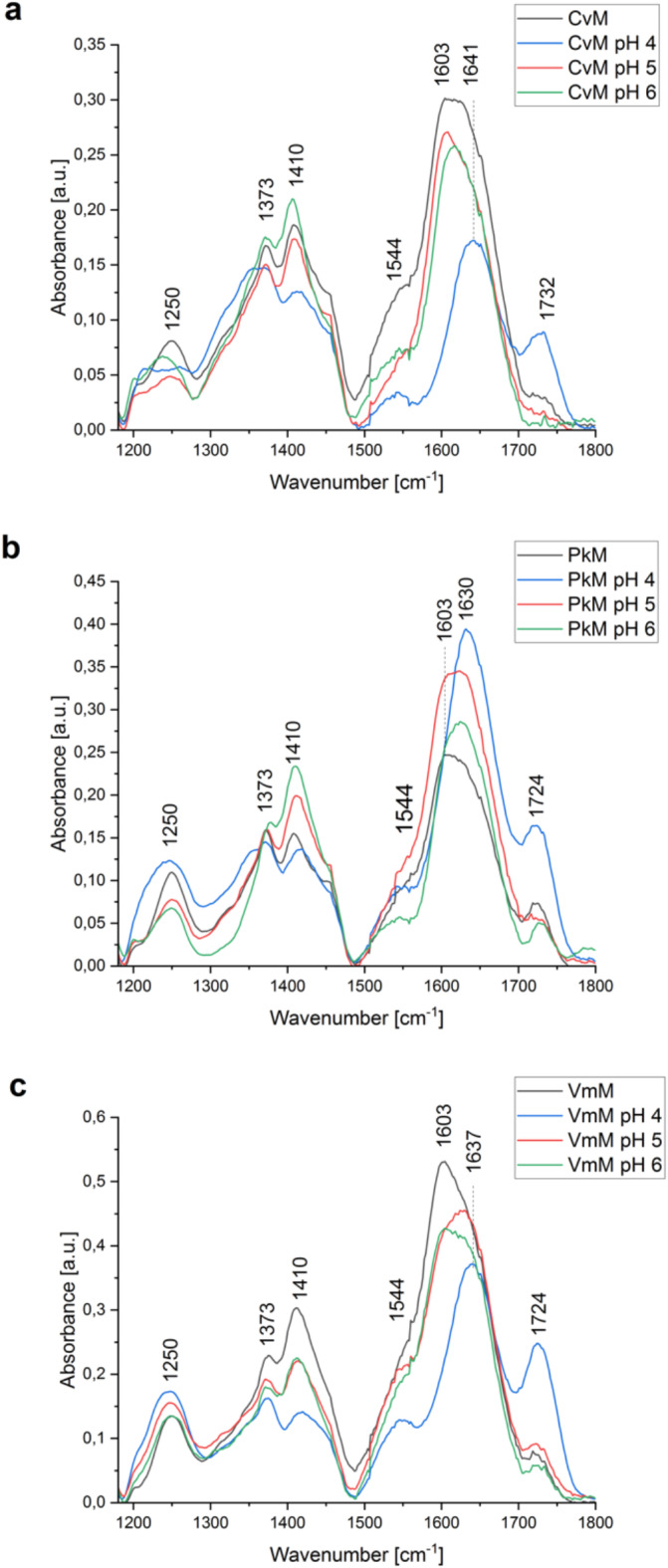
FTIR spectra in the range of 1180–1800 cm^− 1^ of native *Cv*M (**a**), *Pk*M (**b**), and *Vm*M (**c**) and after pH adjustment to pH 4 (blue), 5 (red) and 6 (green).

The analysis of the pH-modified FTIR spectra of *Cv*M, *Pk*M, and *Vm*M was performed to check the effect of the pH value on the functional groups in the studied EPS. The main differences in the FTIR spectrum after the pH modification were visible in the region of 1200–1750 cm^− 1^ (Fig. 1). The bands at ca. 1250 cm^− 1^, 1373 cm^− 1^, 2925 cm^− 1^, and 3290 cm^− 1^ were observed in both the control and pH-modified EPS (Fig. 1). In all the samples tested, the pH value had an impact on the carboxyl group bands (1300–1750 cm^− 1^). It was observed that the intensity of the bands at 1603 cm^− 1^ decreased in *Cv*M and *Vm*M, shifted to approx. 1631–1641 cm^− 1^ at pH 4 in all the EPS samples, and increased in *Pk*M. It was also observed that the bands at ca. 1408–1412 cm^− 1^ shifted to 1416–1421 cm^–1^ at pH 4. The intensity of the band at 1412 cm^− 1^ in *Vm*M decreased at all the pH values as well as the band 1408 cm^− 1^ in the other EPS samples at pH 4. Additionally, a weak band at 1544 cm^− 1^ was observed in all the samples at pH 4, and an increase in the intensity of the band at ca. 1724 cm^− 1^ was recorded.

### Sorption study

**Fig. 2:**
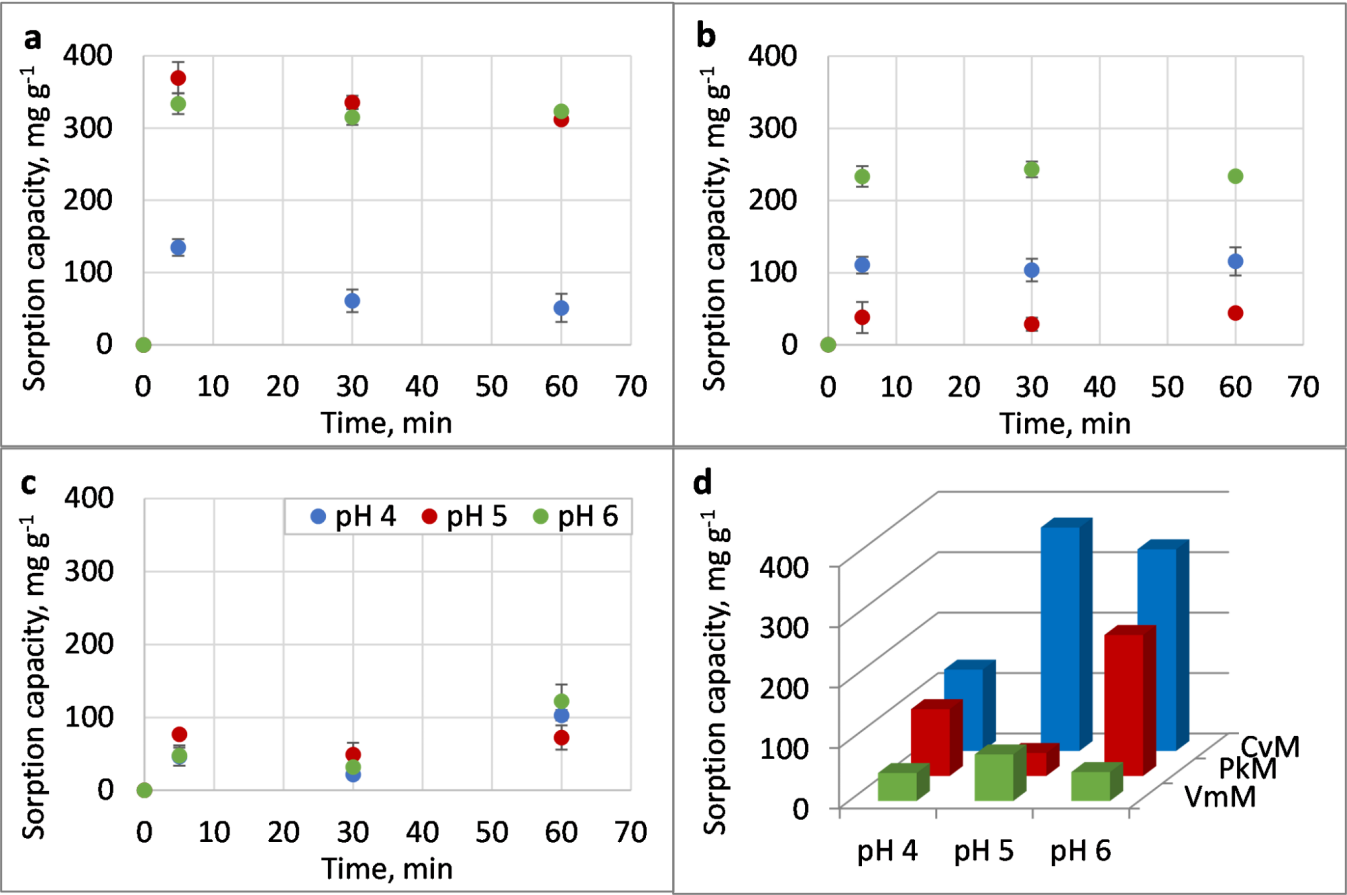
Lead sorption capacity [mg g^− 1^] of *Cv*M (**a**), *Pk*M (**b**), and *Vm*M (**c**) at pH 4 (blue dots), pH 5 (red dots), and pH 6 (green dots) after 5, 30, and 60 min. The graph (**d**) presents the sorption level after 5 min.

 The EPS obtained from the microalgae grown in the mixotrophic conditions were examined to determine their sorption properties towards Pb(II) ions. The effects of the pH value and contact time on the sorption properties of the samples were examined. The results showed that *Cv*M and *Pk*M reached the maximum sorption capacity already within 5 min, followed by stabilisation or desorption (*p* ≤ 0.05) in the case of *Cv*M at pH 4 and pH 5 (Fig. 2a, b). The highest amount of bounded lead ions in *Vm*M was observed after 60 min (Fig. 2c). The results of the sorption capacities of *Cv*M, *Pk*M, and *Vm*M after 5 min are summarised in Fig. 2d.

Among the tested EPS, *Cv*M showed the highest sorption capacity of 369.7 mg g^− 1^ at pH 5 (Fig. 2). The pH 6 value was also found to be suitable for Pb(II) sorption by *Cv*M. However, the sorption properties of *Cv*M decreased at pH 4, and the highest amount of bound ions in these conditions was 134.7 mg g^− 1^. The sorption capacity of *Pk*M was significantly related to the pH value. The highest sorption capacity of *Pk*M ranging between 233 and 243 mg g ^− 1^ was observed at pH 6. The lowest sorption capacity in the range from 28.7 to 43.6 mg g^− 1^ was found at pH 5. At pH 4, the sorption capacity of *Pk*M was between 103.7 and 115.8 mg g^− 1^. In contrast to *Cv*M and *Pk*M, the highest sorption capacity of *Vm*M was recorded at pH 6 after 60 min; it was 122.3 mg g^− 1^ (Fig. 2).

The results of the lead removal potential showed that *Cv*M exhibited the highest Pb(II) removal potential, reaching 50.4% and 50.2% at pH 5 and 6, respectively (Table 3). In turn, the highest Pb removal potential was exhibited by *Pk*M and was in the range of 35–37% at pH 6. The removal potential of the other samples (*Cv*M pH 4, *Pk*M pH 4, *Pk*M pH 5, *Vm*M) was below 20%. It was found in the present study that the sorption capacity of *Pk*M varied significantly depending on the pH value.

**Table 3:** Pb(II) removal potential of *Cv*M, *Pk*M, and *Vm*M.

Removal potential [%]		Contact time		
		5 min	30 min	60 min
*Cv*M	pH 4	17.18 ± 0.11^d^	7.75 ± 1.32^c^	6.48 ± 0.43^c^
	pH 5	50.38 ± 1.43^a^	45.76 ± 0.82^a, b^	42.55 ± 0.43^b^
	pH 6	50.23 ± 4.29^a^	47.43 ± 1.11^a, b^	48.70 ± 0.53^a^
*Pk*M	pH 4	14.10 ± 1.36^b^	12.39 ± 2.78^b^	14.77 ± 2.49^b^
	pH 5	5.22 ± 2.94^a^	3.94 ± 1.23^a^	5.98 ± 0.27^a^
	pH 6	35.85 ± 2.07^c^	37.34 ± 1.57^c^	35.92 ± 0.44^c^
*Vm*M	pH 4	6.63 ± 2.75^a^	3.76 ± 1.78^a^	13.05 ± 0.69^b, c^
	pH 5	12.77 ± 4.15^b, c^	6.64 ± 1.74^a^	8.54 ± 0.14^a, b^
	pH 6	7.31 ± 2.13^a, b^	4.90 ± 0.56^a^	18.61 ± 2.16^c^

The superscript letters in the table indicate statistical significance of the presented results analysed by ANOVA and Tukey’s test (*p* ≤ 0.05) (EPS = 1 mg mL^− 1^; *n* = 3; ± SD).

### FTIR analysis after Pb(II) sorption

**Fig. 3:**
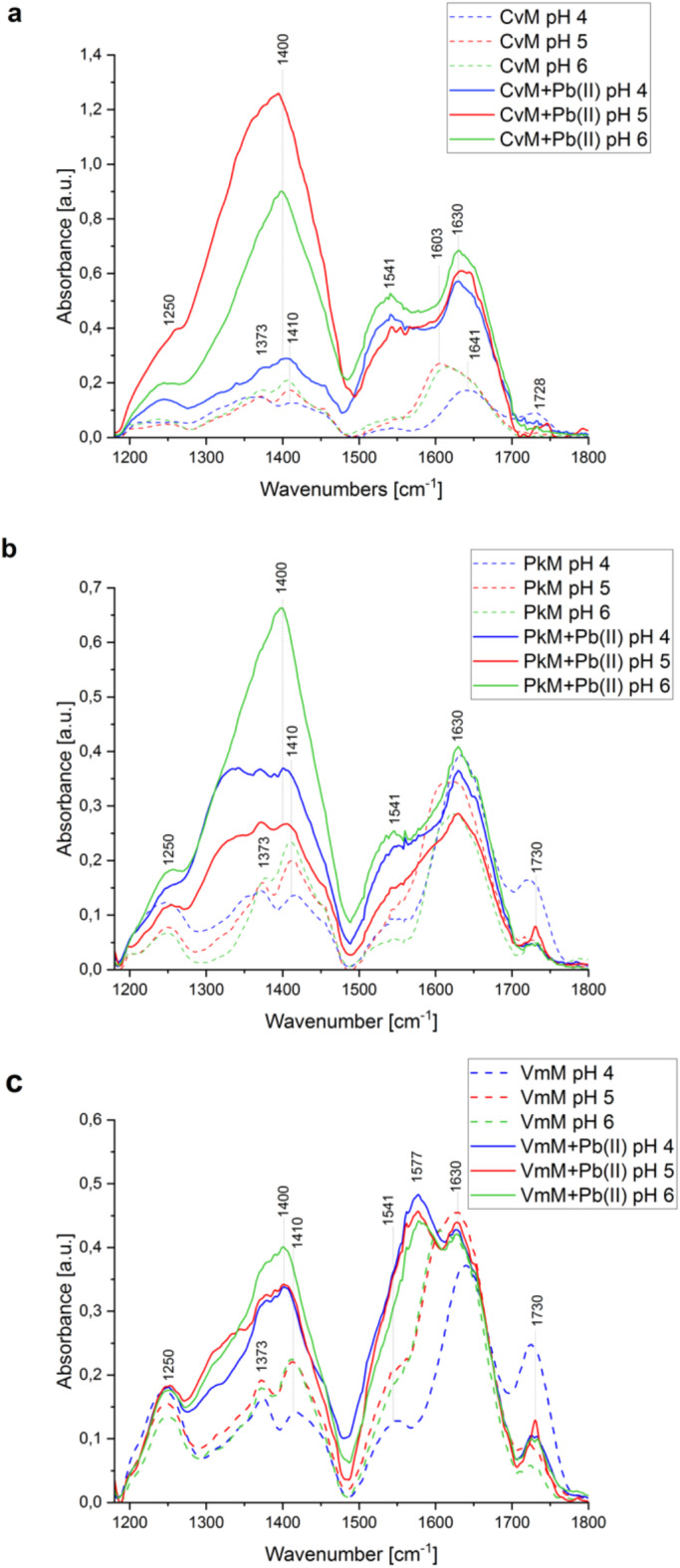
FTIR spectra of *Cv*M (**a**), *Pk*M (**b**), and *Vm*M (**c**) after Pb(II) sorption (solid line) in the range of 1180–1800 cm^− 1^. pH-modified EPS as a control (dash line): pH 4 (blue), pH 5 (red), pH 6 (green).

The interactions of Pb(II) with the studied EPS were analysed using FTIR spectroscopy. In the presence of Pb(II) ions, the bands at ca. 3290 cm^− 1^ shifted to 3278 cm^− 1^, and the intensity of these bands decreased in *Pk*M and *Vm*M and increased in *Cv*M at pH 5 and 6, compared to the pH-modified samples before the Pb(II) sorption (Figure S3). After the sorption of Pb(II) ions, the band at 1728–1730 cm^− 1^ decreased in all the EPS samples at pH 4 and increased at pH 5 (Fig. 3). As shown in Table 4, the bands of carboxyl groups at 1604–1641 cm^− 1^ in the pH-modified EPS were shifted to 1630–1633 cm^− 1^ after the Pb(II) treatment, and the bands from 1408 to 1419 cm^− 1^ were shifted to ca. 1400 cm^− 1^ in all the tested samples. The FTIR spectrum of *Cv*M after the Pb(II) sorption showed an increase in the bands in the range of 1650 –1200 cm^− 1^ (Fig. 3a). An increase in the intensity of the band at 2925 cm^− 1^ was observed at pH 5 in the presence of Pb(II) in all the tested EPS. The intensity of the band at ca. 1544 cm^− 1^ observed in most of the control samples increased in *Cv*M and *Pk*M after the Pb(II) sorption, and in the case of *Vm*M, this band shifted to 1577 cm^− 1^. *Pk*M was also observed to interact with Pb(II) at pH 4 and 5 via the 1329 cm^− 1^ band (Fig. 3b). Additionally, an increase in the intensity of the 3280 cm^− 1^ band was observed in *Cv*M after the Pb(II) sorption, while the intensity of the 3200–3400 cm^− 1^ broad band decreased in the case of *Pk*M and *Vm*M. Moreover, a sharp band at 680 cm^− 1^ was observed in *Cv*M after the Pb(II) sorption at pH 5 and 6 (Figure S3).

**Table 4:** Wavenumbers (cm^− 1^) of the bands of COO^−^ groups in mixotrophic EPS after pH modification and Pb(II) sorption (C_Pb(II)_ = 100 Mg L^− 1^).

Sample	pH 6	pH 5	pH 4	pH 6 + Pb(II)	pH 5 + Pb(II)	pH 4 + Pb(II)
*Cv*M	1410	1412	1419	1398	1400	1404
*Pk*M	1410	1411	1415	1398	1406	1400
*Vm*M	1410	1411	1421	1400	1400	1400
*Cv*M	1618	1608	1631	1630	1630	1630
*Pk*M	1608	1624	1641	1631	1630	1630
*Vm*M	1604	1639	1639	1630	1630	1626

The superscript letters in the table indicate statistical significance of the presented results analysed by ANOVA and Tukey’s test (*p* ≤ 0.05) (EPS = 1 mg mL^− 1^; *n* = 3; ± SD).

## Discussion

The aim of this study was to determine the effect of mixotrophy on the production, chemical composition, and sorption properties of EPS synthesised by unicellular algae. It was found that the EPS yield in the mixotrophic cultures was significantly higher than in the autotrophic ones. Similarly, the total EPS specific productivity was 183.8 mg g^–1^, 118.5 mg g^− 1^, and 56.8 mg g^− 1^ in the case of *Cv*M, *Pk*M and *Vm*M, respectively, in the mixotrophic conditions (Table 1). These values were from three-fold to five-fold higher in comparison to the specific productivity of EPS in the autotrophic growth conditions. The values of the EPS specific productivity were lower than the EPS yield due to the high biomass concentration in the mixotrophic cultures. The results obtained are consistent with those presented by Zhang et al. (2019), who obtained 364.3 mg L^− 1^ and 182.3 mg g^− 1^ of EPS produced by *C. vulgaris* in cultures with the same growth medium composition^14^. Cheirsilp et al. (2016) also reported that *C. vulgaris* cultivated in mixotrophic conditions produced six times more EPS than in autotrophic cultures^2^.

The ability of *V. magna* to produce EPS in autotrophic and mixotrophic conditions is still unexplored in the current literature. It was found in the present study that the total EPS specific productivity in the *V. magna* culture was 16.1 mg g^− 1^ and 56.8 mg g^− 1^ in the autotrophic and mixotrophic conditions, respectively (Table 1). A study of *Vischeria punctata*, a species belonging to the same order, showed EPS production at day 49 ranging between 194 mg g^− 1^ and 253.7 mg g^− 1^^20^. The present study showed the highest yield and specific productivity of total EPS synthesised by *C. vulgaris* cultivated in both the autotrophic and mixotrophic conditions. The results also revealed that the nutritional strategy in unicellular algae exerts an effect on the processes of EPS synthesis by algal cells. Studies on the removal of zinc ions and copper ions by *Tetradesmus obliquus* under autotrophic and mixotrophic cultivation have shown that the addition of glucose (0.2 g L^− 1^ glucose to BG11) stimulated the cells to increase the production of extracellular polymers and thus enhanced metal adsorption^21^. The application of glucose, glycerol, sodium acetate, and sucrose in the mixotrophic culture of *T. obliquus* showed that, among the different carbon sources tested, glycerol, sodium acetate, and sucrose increased the production of EPS^22^. A higher concentration of total soluble polysaccharide than in autotrophic cultures was also reported in a study on *Porphyridium cruentum* microalgae growing in mixotrophic conditions with supplementation of a soluble fraction of potato meal^23^. Studies show that mixotrophic conditions with the use of glucose, galactose, and maltose increased the synthesis of EPS in *N. oleoabundans*^4^. Glucose, fructose, and sucrose are indicated as carbon sources inducing the highest EPS production^24^. The increase in the EPS production in the mixotrophic conditions can be explained by the increase in the carbon to nitrogen (C/N) ratio. This leads to a switch of microalgal metabolism to the increased production of carbohydrates (both extracellular and intracellular)^3^. The availability of carbon in the growth environment is a key factor controlling EPS synthesis, which depends on the type of nitrogen metabolism^25^. Studies on bacterial EPS synthesis indicate that, at a high C/N ratio, bacteria can limit growth and modify their metabolism towards EPS production due to insufficiency of nitrogen required for protein synthesis. However, if nitrogen depletion occurs too quickly before the culture reaches stationary phase, EPS synthesis will also be limited by the small number of cells in the culture. On the other hand, the addition of an organic carbon source to the culture medium can influence the higher requirements of the cells for nitrogen, phosphorus and other elements^26,27^. In conditions of excess carbon, the polysaccharide synthesis process is a result of increased ATP production^24,28,29^. Nutrient stress studies demonstrated that the nitrogen/phosphorus ratio (P/N) also influences the productivity and composition of soluble and bound EPS. Under P-limited conditions and high N concentrations in the wastewater, higher amounts of proteinaceous EPS were found in Chlorella sp. and Micractinium sp. cultures^30^. Boonchai et al. (2015) showed that more EPS, both bound and soluble, was extracted under nitrogen and phosphorus deficient conditions. Soluble EPS was characterised by higher amounts of proteins than control samples under conditions with sufficient nitrogen and phosphorus concentrations^31^.

The presence of organic carbon in the growth medium exerts an effect on the metabolism of microalgae and the chemical composition of their EPS. The present results indicate that the mixotrophic growth conditions enhanced the carbohydrate content in the EPS. The content of total sugars in the EPS was 86.5%, 90.6%, and 72.4% in *Pk*M, *Cv*M, and *Vm*M, respectively. The content of total sugars in the EPS of the mixotrophic samples was about twice as high as in the *Pk*A and *Vm*A samples from the autotrophic conditions (Table 2). An increase in carbohydrate content in EPS produced in mixotrophic conditions was also observed by Vo et al., who showed the highest increase in sugar content in *C. vulgaris* EPS in the presence of glucose as an organic carbon source^32^. The sugar content in EPS synthesised by *P. kessleri* in autotrophic conditions varied from 82 mg g^− 1^^33^ to approximately 738 mg g^− 1^ (73.8 weight%)^34^, and the concentration of total sugar in *C. vulgaris* EPS ranged from 495.4 mg g^− 1^^16^ to approximately 670 mg g^− 1^ (67 weight%)^35^. In the current study, the content of total sugars in *Vm*A and *Vm*M was 36.9 weight% and 72.4 weight% (369 and 724 mg g^− 1^), respectively (Table 2). For comparison, the sugar content in other Eustigmatophyceae species was 247.5 mg g^− 1^ of neutral sugars in *Vischeria punctata* EPS^20^ and 31 mg g^− 1^ in *Nannochloris* sp. *Naumann* EPS^36^. In the mixotrophic conditions, the content of reducing sugars also increased (Table 2).

The current study indicated that the mixotrophic growth conditions increased the content of proteins and amino sugars in the EPS and the content of amino acids in the EPS synthesised in *C. vulgaris* and *V. magna* (Table 2). It has been reported that mixotrophic growth conditions lead to increased protein accumulation in microalgal cells^37,38^, which may have an impact on the protein content in EPS, and may be a source of amino groups. The literature data on the amino acid content in EPS produced by microalgae are limited, although there are available data on EPS produced by diatoms and *Porphyridium cruentum*^39–41^. In contrast, the amino acid content determined in the bacterial extracellular proteoglycan produced by *Rhodococcus rhodochrous* was 113.12 µg mg^− 1^^42^. In turn, the amount of uronic acids decreased in the mixotrophic samples from 14.5 weight% of uronic acids in the EPS from the autotrophic culture to 13.5 weight% in the EPS from the mixotrophic culture. The amount of uronic acids in *Cv*A as well as *Pk*A corresponds to the results obtained by Ogawa, Ikeda, and Kondo^43^. Uronic acids are the most often studied EPS components, as they play a key role in metal complexation and flocculation processes^5^. Uronic acids provide negatively charged functional groups that can interact with metal ions, which underlies their importance in the sorption properties of EPS. Reduction in the uronic acid content may decrease the sorption capacity of EPS and affect its surface charge^5,11^.

The results showed a positive effect of the addition of organic carbon to the algal culture media on the content of phenolic compounds in the EPS of all the studied species. These findings suggest a better radical scavenging capacity of the EPS produced in the mixotrophic conditions^44,45^. The content of phenolic compounds in *Cv*A and *Vm*A was similar to that in EPS produced by *C. vulgaris* in other studies^46^.

The analysis of the monosaccharide composition indicated that *Cv*A and *Pk*A comprised mostly rhamnose, fructose, and galactose, while *Vm*A was mainly composed of rhamnose and galactose. In turn, *Cv*M, *Pk*M, and *Vm*M produced stronger mannose and glucose spots. *Vm*A did not contain fructose, unlike *Pk*A and *Cv*A. The results of the analysis of the EPS synthesised by *C. vulgaris* and *P. kessleri* indicate that the similarity in the monosaccharide composition in the EPS may result from the phylogenetic relationship between these microalgae (family Chlorellaceae). Mixotrophic EPS from *C. vulgaris* studied by Zhang et al. was shown to consist mostly of galactose (45.4%) after 5 days of culture^14^. Allard and Tazi observed a decrease in galactose content in EPS produced by *Chlamydomonas augustae* during the culture period, while the amount of glucose and glucuronic acid increased^47^. This suggests that the duration of the culture may have an impact on the monosaccharide composition of EPS^47^. In the current study, the culture was carried out for 25 days. The presence of rhamnose, mannose, galactose, and glucose was also detected in other EPS obtained from *C. vulgaris* cultured in mixotrophic conditions^14^, while fructose and xylose were identified in EPS samples from autotrophic *C. vulgaris* cultures^35,48^. Mixotrophic EPS produced by *Parachlorella* sp. BX1.5 was mainly composed of rhamnose (48.5 mol%), but glucose, galactose, and xylose were identified as well^34^. The results are in contrast to these obtained in this study, where *Pk*M consisted mostly of glucose, while rhamnose predominated in *Pk*A. The autotrophic *P. kessleri* EPS was also reported to be dominated by rhamnose in addition to mannose and galactose^1^. The use of other organic carbon sources results in different EPS production, carbohydrate and protein content^32^ and monosaccharide composition^49,50^. However, the chemical composition of EPS is known to change during cultivation^47^ and is also susceptible to environmental changes^5^. Some environmental factors cause exopolymer degradation, such as temperature, acid pH or enzymes with anti-biofilm activity^51^. For example, environmental acidification can lead to folding and aggregation of EPS, reducing charge density and therefore sorption potential^52^. Additional studies are needed to design experiments that can monitor long-term dynamics, as this will be crucial to further understanding the phenomena under investigation.

The analysis of the FTIR spectra of the *Cv*A, *Pk*A, and *Vm*A indicated the presence of hydroxyl (3275–3346 cm^− 1^), CH_2_/CH_3_ (2850 cm^− 1^, 2919–2935 cm^− 1^), carboxyl (1394–1737 cm^− 1^), and phosphate or sulphate groups (1238–1259 cm^− 1^) and a sugar fingerprint (1016–1037 cm^− 1^) (Figure S2, Table S1). The EPS synthesised in the mixotrophic conditions was characterised by a decrease in the intensity of bands of carboxyl, and phosphate, or sulphate groups in all the tested samples (Figure S2). The modification of the pH value in the *Cv*M, *Pk*M, and *Vm*M resulted in shifts within the bands of symmetric (~ 1410 cm^− 1^) and asymmetric (~ 1604 cm^− 1^) vibrations of carboxyl groups (Table 4). The reduction of the pH value to 4 also resulted in the appearance of C=O vibrations in the -COOH group at 1724 cm^− 1^ (Fig. 1)^53^. The observed changes may be a result of protonation of carboxyl groups^54^ or interactions with cations present in the EPS^53^. Moreover, the decrease in the intensity of the carboxyl group band at ca. 1410 cm^− 1^ and ca. 1604 cm^− 1^ may also be a result of protonation of -COO^−^ groups, as the intensity of -COOH vibrations increased (1724 cm^− 1^) at pH 4 for all the samples (Fig. 1)^55^. Acidic pH has also been shown to affect the spatial organisation of EPS, increasing its aggregation^56^. In turn, Burgain et al. (2015) observed EPS folding at pH 4.8 (in opposite to the unfolded structure at pH 6.8), resulting in a lower charge density at acidic pH and a thinner EPS layer^57^.

Natural environments or wastewater are very complex systems. In order to understand the course of the sorption process in the environment, it is necessary to experimentally investigate the course of this process under laboratory conditions, taking into account different combinations of factors. In this work, the influence of mixotrophic conditions and pH value on the amount of Pb ions associated by the tested EPS was analysed. The results of the sorption study showed the highest sorption efficiency of *Cv*M and *Pk*M after 5 min of contact time, which is consistent with the result obtained by Ghorbani et al. (2022), who reported sorption equilibrium after 10 min, followed by slight desorption^58^. The amount of lead ions sorbed by *Cv*M and *Pk*M (335.8 mg g^− 1^ and 28.7 mg g^− 1^, respectively) was lower than values obtained for EPS synthesised in autotrophic conditions reported in a previous study^1^. *Vm*M showed the lowest sorption capacity in the studied parameters, as the amount of Pb(II) bonded by this polymer did not exceed 20 mg g^− 1^. This result is higher than that observed for the capsular polysaccharide from *Gloeocapsa gelatinosa*, which was found to adsorb 8.59 mg g^− 1^ Pb(II)^59^. The pH value is a key factor affecting the metallic ion solution as well as the biosorbent functional groups^60^. It was found in the present study that the Pb(II) removal potential of the EPS synthesised by *P. kessleri* reached the maximum value at pH 6.0 for all the contact times tested. The present study is in line with the findings reported by Wei et al. (2016), who found the maximum Pb(II) adsorption capacity (99.47 mg g^− 1^) of EPS extracted from *Klebsiella* sp. J1 at pH 6.0^60^. In the case of *Vm*M and *Cv*M, the maximum Pb(II) removal potential after the 5-min contact time was achieved at pH 5 and after 60 min also at pH 6. It was found that the lowest values of the Pb(II) removal potential by *Cv*M were obtained at pH 4. The reason may be the increase in the share of the protonated form of carboxyl groups (COOH) (Fig. 1)^61^. The degree of protonation of functional groups influences the sorption properties of the polymer by blocking or releasing binding sites. At acidic pH, Pb ions compete with H^+^ for the binding sites, which reduces sorption efficiency^62^. The low adsorption at the low pH value is related to an increased positive charge density at the EPS surface, which causes electrostatic repulsion between metal ions and positively charged groups^63^. For example, amino groups (NH_2_) at low pH turn into the cationic form (NH_3_^+^); hence, electrostatic repulsion forces occur and prevent Pb(II)–EPS interactions^63^. As the pH value increases, the competition from hydrogen ions decreases and the functional groups of EPS dissociate and become negatively charged (COOH → COO^–^), resulting in adsorption to the EPS surface^60,62^. The other point was presented by Adeniran (2024), who observed improved sorption efficiency at pH 2–5, which is in contrast to the results obtained in this paper^64^. The current findings indicate that the EPS produced by *C. vulgaris* in the mixotrophic conditions has higher lead removal potential than the *P. kessleri* and *V. magna* EPS at the three pH value tested.

After Pb(II) sorption, the band at 1730 cm^− 1^ disappeared, indicating an ion exchange interaction of Pb ions with the -COOH groups (Fig. 3)^53^. The presence of lead ions shifted the bands of the -COO^−^ groups to 1630 cm^− 1^ and ca.1400 cm^− 1^ in all the tested samples (Table 4). This may suggest complexation of Pb(II) by the -COO^−^ groups^53,65,66^. The weak band of carboxyl groups at 1544 cm^− 1^ observed in the mixotrophic EPS (also pH-modified) increased its intensity and, in the case of *Vm*M, shifted to a further wavenumber (1577 cm^− 1^)^67^. After the Pb(II) treatment, the broad band of -OH groups at ca. 3300 cm^− 1^ was shifted to the sharpened 3278 cm^− 1^ band (Figure S3). The involvement of hydroxyl groups in the EPS–Pb(II) interaction was particularly evident in *Cv*M^68^. The sharp band at 680 cm^− 1^ indicated the presence of Pb–O–Pb bonds (Figure S3a)^69^.

*Cv*M after Pb(II) sorption at pH 5 and 6 as well as *Pk*M–Pb(II) at pH 6 showed the highest intensity of the 1400 cm^− 1^ band, which corresponded to the highest amount of Pb ions bound by EPS after 60 min (Figs. 2a–c and 3). In turn, *Pk*M and *Vm*M exhibited the lowest Pb(II) sorption at pH 5 after 60 min and the highest intensity of C–H vibrations (2850–2924 cm^− 1^ and 1329 cm^− 1^)^70^ as well as an increase in the band of COOH groups (1730 cm^− 1^) (Fig. 3, Figure S3). The participation of -COO^−^ groups (1400 cm^− 1^) in the Pb(II) sorption by *Vm*M, *Pk*M pH 5, and *Cv*M pH 4 was comparable and corresponded to the lowest amount of bound metal ions. Future research should provide additional structural information to better understand the Pb(II)-EPS interaction.

Mixotrophy increases the productivity of biomass and exopolymer, thus improving the applicability of both products. The ability to utilise organic carbon predisposes the investigated species to be used in the treatment of wastewater containing organic carbon. In turn, the ability to remove lead may allow the use of microalgae in wastewater contaminated with heavy metals.

According to the literature, EPS can be used to remove micropollutants as well as ammonium or phosphate ions from the environment^6,71–73^. In order to consider the environmental application of the isolated EPS, the research needs to include more factors that would simulate natural conditions. The interaction between metal and EPS is also influenced by other elements, nutrients or other environmental factors^10^.

In the present study, EPS produced by *C. vulgaris*,* P. kessleri*, and *V. magna* in mixotrophic conditions was investigated. The highest productivity and yield of total and soluble EPS were found in the mixotrophic *C. vulgaris* cultures. The compositional analysis of the soluble fractions of the tested exopolymers revealed that they were mainly composed of carbohydrates. The addition of glucose to the culture medium causes changes in cellular metabolism, resulting in changes in the chemical composition of the EPS – in particular, to an increase in protein and carbohydrate content. FTIR spectroscopy indicated that carboxyl and hydroxyl groups are the major contributors to Pb(II) sorption. Among the tested samples, EPS produced by *C. vulgaris* revealed the highest Pb(II) removal potential and sorption capacity at pH 5 and 6 for all the contact times tested.

## Materials and methods

### Cultured strains and algal culture conditions

*Chlorella vulgaris* CCALA 788 and *Parachlorella kessleri* CCALA 250 were obtained from the Culture Collection of Autotrophic Organisms (Třeboň, Czech Republic). *Vischeria magma* SAG 36.89 (*Eustigmatos magnus*) was purchased from the Culture Collection of Algae at Goettingen University (SAG). Inocula were cultivated in Erlenmeyer flasks containing sterile BG-11 medium in a 16 h : 8 h light : dark system at the light intensity of 60 µmol m^− 2^ s^− 1^ on a rotary shaker with aeration with sterile air.

### Experimental culture

 The autotrophic culture of *C. vulgaris*, *P. kessleri*, and *V. magma* was carried out on BG-11 medium. For mixotrophic cultivation of the analysed strains, the BG-11 medium was supplemented with 5 g L^− 1^ of glucose according to literature^14^. The growth media were inoculated to initial culture optical density 0.2. The cultures were carried out for 25 days in glass photobioreactors with a working volume of 3 L with aeration with sterile air. The microalgae were cultivated in a 16 h : 8 h light : dark system at the light intensity of 60 µmol m^− 2^ s^− 1^ and a temperature of 23 ± 1 °C. Each experimental culture variant was carried out in three replications.

### Measurement of biomass concentration

 Biomass concentration was monitored by measuring biomass dry weight (DW). For this purpose, the algal cells were filtered through Whatman GF/C glass filters (Cytiva, Maidstone, UK) and then dried in a laboratory drier to constant weight and weighed.

### EPS isolation and productivity calculation

 The microalgal biomass was removed from the culture medium by centrifugation at 12,610 x g for 30 min at 4 °C. The supernatant was then filtrated under reduced pressure to remove residual cells and concentrated using a rotary evaporator (Heidolph, Germany). EPS were isolated from the concentrated culture medium using the ethanol precipitation method. Two volumes of 96% cold ethanol were added to the concentrated solution. The mixture was stirred and left for 72 h at 4 °C. After this time, the mixture was centrifuged (12610 x g, 30 min, 4 °C), and precipitated EPS was dissolved in demineralised water, dialysed using a cellulose membrane with a 12−14 kDa cut-off (Bionovo, Legnica, Poland), and freeze-dried (Labconco, Kansas City, MO, USA). The obtained EPS was redissolved in demineralised water and centrifuged after 24 h. The supernatant containing soluble EPS was freeze-dried again^1,42^. The yield [mg L^− 1^] and specific productivity [mg L^− 1^] of the EPS from *Cv*A (EPS synthesised by *C. vulgaris* in the autotrophic conditions), *Cv*M (EPS synthesised by *C. vulgaris* in the mixotrophic conditions), *Pk*A (EPS synthesised by *P. kessleri* in the autotrophic conditions), *Pk*M (EPS synthesised by *P. kessleri* in the mixotrophic conditions), *Vm*A (EPS synthesised by *V. magna* in the autotrophic conditions), and *Vm*M (EPS synthesised by *V. magna* in the mixotrophic conditions) were calculated according to equations described in a previous study^1^.

## Biochemical composition

### Carbohydrate and protein content in EPS

 For the analysis, the freeze-dried EPS was dissolved in demineralised water to a concentration of 1 mg mL^− 1^. The total sugar content was measured with the phenol-sulphuric method. Culture samples were first centrifuged to remove microalgal cells. For the measurement, 200 µL of the culture medium was taken and then sulphuric acid and a 5% aqueous phenol solution were added. After 5 min of incubation at 90 °C, the total sugar concentration was measured using a Cary 300 UV-Vis spectrophotometer (Varian Medical Systems, Inc., Belrose, NSW, Australia) at 490 nm with 1 mg mL^− 1^ glucose (POCH, Gliwice, Poland) as a standard^74^. The protein concentration was determined according to the Bradford method, using 1 mg mL^− 1^ of bovine serum albumin (Sigma Aldrich, St. Louis, MO, USA) as a standard^75^.

### Total phenolic content in EPS

 The spectrophotometric method with diazosulfanilamide (DASA) was used to evaluate the content of phenolic compounds in the EPS. The test sample was mixed with 0.2 mL of a 1% DASA solution in 10% HCl and then with 0.2 mL of 5% NaNO_2_. The mixture was left for 5 min, after which 1 mL of 20% Na_2_CO_3_ was added. The absorbance was measured spectrophotometrically at 500 nm. The concentration of phenolic compounds was determined using vanillic acid as a standard^76^.

### EPS hydrolysis

 The EPS samples were hydrolysed by 4 M trifluoroacetic acid (TFA) (Sigma-Aldrich, St. Louis, MO, USA) (temperature = 100 °C, time = 4 h). The EPS hydrolysis was performed in a thermoblock (Macherey-Nagel, Duren, Germany). After hydrolysis, TFA was removed from the samples through evaporation. Then, the hydrolysed samples were dissolved in demineralised water and evaporated. The procedure was repeated three times to obtain preparations for further analysis^42^.

### Assessment of reducing sugars in EPS

 In order to determine sugars in the EPS, the content of reducing sugars and uronic acids was quantified. The concentration of reducing sugars was determined with the Somogyi-Nelson method, where the absorbance was measured spectrophotometrically at 520 nm using glucose as a standard (POCH, Gliwice, Poland)^77^.

### Assessment of uronic acids in EPS

 The content of uronic acids was determined using the carbazole-sulphuric method. The product of the condensation reaction of uronic acid anhydrides with carbazole was measured colorimetrically at 530 nm with galacturonic acid (Sigma Aldrich, St. Louis, MO, USA) as a standard^78^.

### Determination of amino acids in EPS

 A reaction with ninhydrin reagent was performed for the determination of the amino acid content. The resulting purple-blue product was quantified spectrophotometrically at 570 nm using glycine as a standard (POCH, Gliwice, Poland)^79^.

### Determination of amino sugars in EPS

 The total amount of amino sugars was determined using a modified Elson-Morgan method. Amino sugars were first acetylated, which was followed by the addition of 99.5% cold ethanol and a 4-(dimethylamino)benzaldehyde solution in HCl. The absorbance of the reaction product was measured spectrophotometrically at 530 nm. D-glucosamine was used as a standard^80^.

### Thin-layer chromatography (TLC) of monosaccharides

 Thin-layer chromatography was employed for the determination of the composition of monosaccharides. For this purpose, 30 µL of hydrolysed EPS and standard solutions were transferred to silica gel 60F_254_ plates (Merck, Germany) and dried. For identification of monosaccharides, standard solutions of glucose, galactose, mannose, fructose, xylose, and rhamnose (Sigma, USA) were used at a concentration of 1 mg mL^− 1^. The plates were placed in a chromatographic chamber pre-saturated with a developing phase consisting of 1-propanol : ethyl acetate : water (4:0.5:0.5 v/v). The separation was carried out for 4 h. Then, the plates were dried, sprinkled with 10% H_2_SO_4_ in ethanol, and heated at 100 °C for 15 min to visualise separated monosaccharides^81^.

### Sorption experiment

 A solution of lead ions was prepared by dissolving Pb(NO_3_)_2_ (Chempur, Poland) to the Pb(II) concentration of 100 mg L^− 1^. For the experiment, an EPS sample was dissolved in a Pb(II) solution to the final concentration of 100 mg L^− 1^. Then, the pH of the samples was adjusted to 4, 5, and 6, respectively. The samples were placed on a rotary shaker with parameters set at 120 rpm and 25 °C. The samples were collected after 5, 30, and 60 min and centrifuged at 9500 rpm for 12 min (Rotanta 460 RS, Hettich, Germany). The supernatant was filtered using 0.45 μm PTFE filters (Chemland, Poland) and intended for metal concentration measurements using optical emission spectrometry with inductively coupled plasma (ICP-OES). The control EPS samples were dissolved in demineralised water to a concentration of 100 mg L^− 1^ with pH set to 4, 5, and 6, centrifuged, and filtered. After the experiment, the pH-modified EPS were freeze-dried for the FTIR analysis.

### ICP-OES measurement

 The concentration of Pb(II) ions in the filtrates after the sorption kinetics experiment was measured using ICP-OES iCAP 6500 Duo (Thermo Fisher Scientific, Waltham, MA, USA) according to Ciempiel et al. The lead concentration was measured using the 220.353 (nm) wavelength. The removal potential and the sorption capacity were calculated following formulas described previously^1^.

### Collection and manipulation of Fourier transform infrared (FTIR) spectra

 The FTIR spectra were recorded with a Nicolet 6700 FTIR spectrometer (Thermo Scientific, USA) equipped with an attenuated total reflection (ATR) attachment. The spectra were collected in the range of 4000–400 cm^− 1^ at a 4 cm^− 1^ interval. The resolution of the spectra was 128 scans. Baseline correction of the spectra was performed using OMNIC software (v.8.2, Thermo Fischer Scientific Inc., Madison, WI, USA)^38^. The spectra were normalised to 1.0 at 1022–1036 cm^− 1^ using MS Excel 2017 (Microsoft Co, Redmond, WA, USA). Further, the spectra were analysed using Origin Pro 2024b (OriginLab Co., Northampton, MA, USA).

### Statistical analysis

 The data were analysed using one-way ANOVA and Tukey’s post-hoc test (*p* ≤ 0.05). The normal distribution of the data analysed was checked using the Shapiro-Wilk test. The Brown-Forsythe test was performed to ensure homogeneity of variance. On the basis of the analysis performed (multiple comparison test and analysis of variance), one-way analysis of variance (ANOVA) was chosen for the analyses presented, due to a more accurate representation of statistical significance. The analysis was consisted of comparing the means of the groups studied, followed by a post-hoc test. Then, one-way analysis of variance (ANOVA) was performed, which consisted in comparing the means of the groups studied, followed by a post-hoc analysis. All statistical analyses were performed using STATISTICA 13.1 software (StatSoft Inc., Tulsa, USA). Measurements in each experiment were performed in three replications.

## Electronic supplementary material

Below is the link to the electronic supplementary material.

Supplementary Material 1

## Data Availability

The datasets used and analysed during the current study available from the corresponding author on reasonable request.
