# Supplementary material for: Lead biosorption and chemical composition of extracellular polymeric substances isolated from mixotrophic microalgal cultures
Source: Sci Rep. 2025 Mar 17;15:9093. doi: 10.1038/s41598-025-94372-9 (PMC11914501; doi:10.1038/s41598-025-94372-9)
Supplement: Supplementary file 1 — Supplementary Material 1 [file 41598_2025_94372_MOESM1_ESM.docx]

Lead biosorption and chemical composition of extracellular polymeric substances isolated from mixotrophic microalgal cultures

Wioleta Ciempiel^a^, Magdalena Czemierska^b^, Dariusz Wiącek^a^, Marlena Szymańska^a^,

Anna Jarosz-Wilkołazka^b^, Izabela Krzemińska^a,*^

^a^ Institute of Agrophysics, Polish Academy of Sciences, Doświadczalna 4, 20-290 Lublin, Poland

^b^ Department of Biochemistry and Biotechnology, Institute of Biological Sciences, Maria Curie-Skłodowska University, Akademicka 19, 20-033 Lublin, Poland

*Corresponding author: i.krzeminska@ipan.lublin.pl, Tel: + 48 (81) 744 50 61

Key words: exopolysaccharides, microalgae, mixotrophy, FTIR, sorption, metal removal


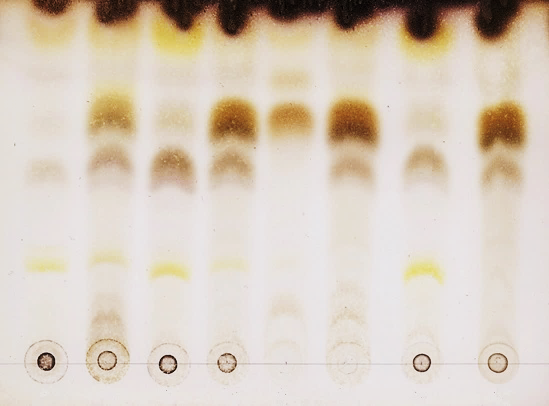

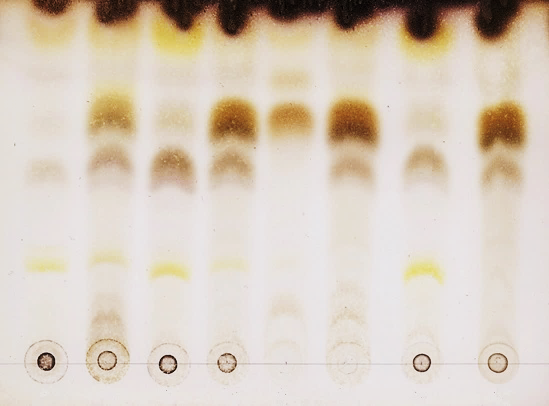


*Vm*A *Vm*M *Cv*A *Cv*M *Pk*A *Pk*M

Rha

Xyl

Man

Glc

Fru

Gal

X


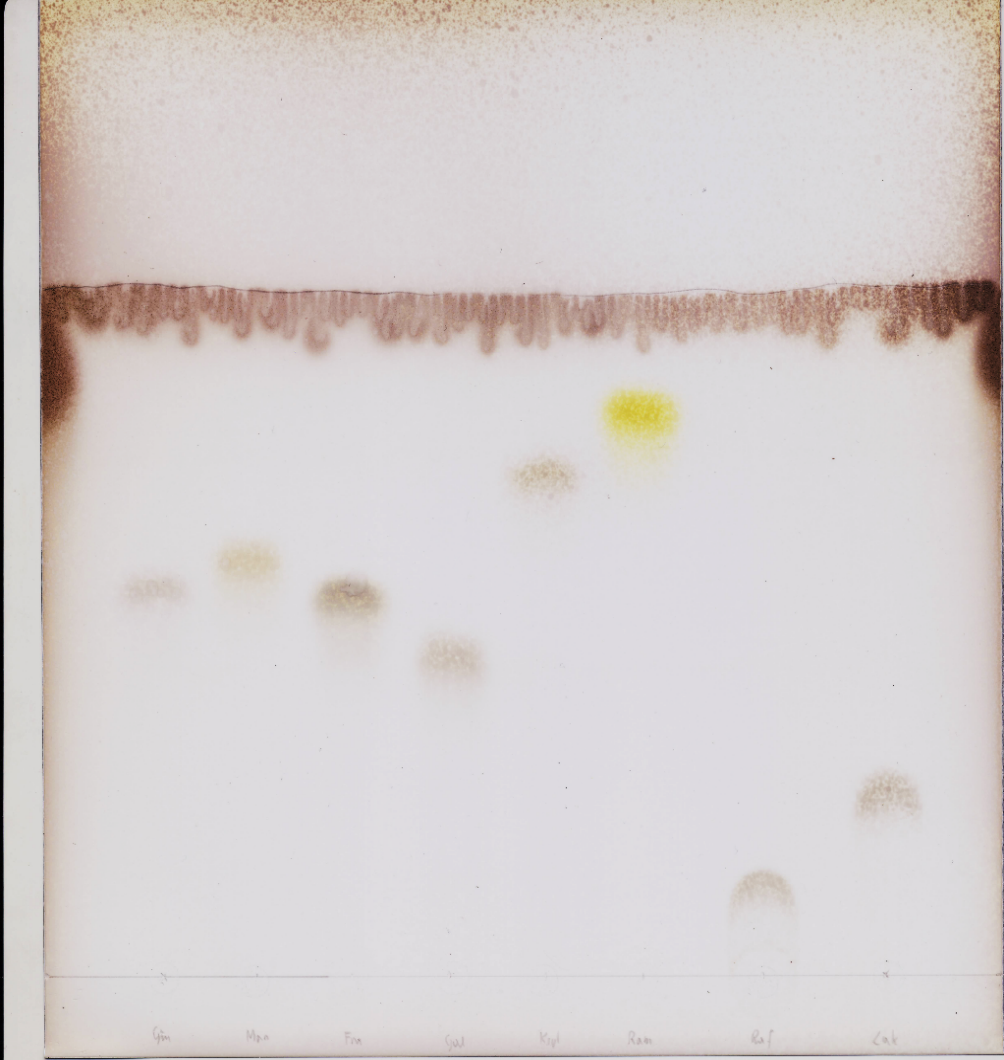


Glc Man Fru Gal Xyl Rha

**a**

**b**

**Figure S1**. TLC plates showing the monosaccharide composition of EPS (a) and standards monosaccharide (b).

**Figure S2**. FTIR spectra of autotrophic (black line) and mixotrophic (red line) EPS samples of *C. vulgaris* (a), *P.* *kessleri* (b) and *V. magna* (c) in the range 600-3800 cm^–1^.

**Figure S3.** FTIR spectra *Cv*M (a), *PkM* (b) and *VmM* (c) after Pb(II) sorption process in the range 600-3800 cm^–1^ (the region 1800–2500 cm^–1^ was removed due to the lack of spectral features).

Table S1. FTIR wavenumbers assignment.

| Wavenumber (cm^-1^) | Assignment | Interpretation | Assignment | Reference |
| --- | --- | --- | --- | --- |
| 781-800 | (CO), δ(COC), (CH) | furanose and pyranose  rings of saccharides,  β -glycosidic bonds | Sugar ring (mannose, galactose) | Fernando et al., 2017 |
| 820-831 | (CO), (CH)  γ(C-OH)ring | furanose and pyranose  rings of saccharides | Sugar ring (annuronic unit) | Fernando et al., 207,  Gawkowska et al., 2018 |
| 1016-1037 | ν(CO), (CC), (COH), | glycosidic bonds | Sugar ring | Sandula et al., 1999, Papageorgiou et al., 2010 |
| 1136-1140 | ν(COC)  CO, CC  C-OH | glycosidic bonds | Sugar ring (glucose unit) | Sandula et al., 1999,  Frenando et al., 2017 |
| 1238-1259 | ν_as_(PO), *ν*_as_(SO), | asymmetric stretching of P═O and S═O | phosphate and sulfate group | Alam et al., 2014, Frenando et al., 2017 |
| ~1330 | δ (OH), δ (CH) | bending of O-H groups in pyranose ring of pectins, bending vibration of the C-H in the ring | CH, OH | Szymańska-Chargot et al., 2015, Gawkowska et al., 2018 |
| 1371-1379 | ν(CO)  δs(CH) | CO stretching,  C-H vibrations and CH_2_ bending | CO, -CH, -CH_3_ | Szymańska-Chargot et al., 2015, Gieroba et al., 2023 |
| 1394-1419 | ν_s_(COO) | non-esterified carboxyl groups | -COO^–^ | Papageorgiou et al., 2010 |
| 1541-1577 | ν_as_(COO) | vibrations of C-O band in COOR group | -COO^–^ | Gawkowska et al., 2018 |
| 1597-1616 | ν_as_(COO) | non-esterified carboxyl groups | -COO^–^ | Szymańska-Chargot et al., 2015, Papageorgiou et al., 2010 |
| 1624-1641 | (COO), (HOH) | ring carboxyl groups,  bound water | COOH, H_2_O | Gieroba et al., 2023,  Szymańska-Chargot et al., 2015, |
| 1722-1747 | ν(C=O) | esterified carboxyl groups | COOH | Szymańska-Chargot et al., 2015 |
| 2850 | ν_as_(CH) | hydrocarbon bond | CH | Gieroba et al., 2020 |
| 2916-2935 | ν_as_(CH) | hydrocarbon bond | CH_2_, CH_3_ | Gieroba et al., 2023 |
| 3275-3346 | ν(OH) | hydroxyl groups | OH | Frenando et al., 2017 |
